# Establishing Sprinkling Requirements on Trailers Transporting Market Weight Pigs in Warm and Hot Weather

**DOI:** 10.3390/ani4020164

**Published:** 2014-04-11

**Authors:** Rebecca Kephart, Anna Johnson, Avi Sapkota, Kenneth Stalder, John McGlone

**Affiliations:** 1 Department of Animal Science, Iowa State University, Ames, IA 50011, USA; E-Mails: rkdavis@iastate.edu (R.K.); stalder@iastate.edu (K.S.); 2 Departments of Animal and Food Science and Animal Care Services, Texas Tech University, Lubbock, TX 79409, USA; E-Mails: asapkota@purdue.edu (A.S.); john.mcglone@ttu.edu (J.M.)

**Keywords:** market-weight pig, sprinkling, transport loss, well-being

## Abstract

**Simple Summary:**

Transport is an inevitable process in the modern, multi-site swine industry. Pigs do not have efficient physiological means (such as sweating) to cool themselves. Therefore, being transported in hot weather can cause heat stress and even death. Sprinkling the pigs and/or bedding may facilitate cooling, thereby improving well-being and survivability of pigs arriving at the plant.

**Abstract:**

This study was conducted July of 2012 in Iowa, in WARM (<26.7 °C) and HOT (≥26.7 °C) weather. Four sprinkling methods were compared, with one treatment being randomly assigned to each load: control- no sprinkling (not applied in HOT weather), pigs only, bedding only, or pigs and bedding. Experiment 1 used 51 loads in WARM- and 86 loads in HOT weather to determine sprinkling effects on pig measures (surface temperature, vocalizations, slips and falls, and stress signs). Experiment 2 used 82 loads in WARM- and 54 loads in HOT weather to determine the sprinkling effects on transport losses (non-ambulatory, dead, and total transport losses). Experiment 1 found that, in WARM weather, there were no differences between sprinkling treatments for surface temperature, vocalizations, or slips and falls (*p* ≥ 0.18)*.* However, stress signs were 2% greater when sprinkling pigs- or bedding only- compared to control (*p* = 0.03). Experiment 2 found that, in WARM and HOT weather, sprinkling did not affect non-ambulatory, dead, or total transport losses (*p* ≥ 0.18). Although the current study did not find any observed sprinkling effects for pig measures or transport losses it is extremely important to note that the inference space of this study is relatively small, so further studies should be conducted to see if these results are applicable to other geographical regions and seasons.

## 1. Introduction

Transporting swine is essential to the multi-site pork production. Around 113 million pigs were marketed in 2012 in the U.S. [1]. For pigs, marketing is a combination of potentially novel (defined as the first exposure), unfamiliar (defined as more than one exposure that is infrequent), and physically exerting experiences that could be perceived as stressful [2]. The term “*transport losses*” refers to pigs that become non-ambulatory (unable to keep up with the group and may have a structural injury) or are dead on arrival [2]. Increased transport losses and decreased meat quality may result if the pig is unable cope with these stressors [3,4,5]. 

The conditions under which pigs are handled and transported can have a direct impact on the pigs’ well-being. In the U.S., trailers rely on passive ventilation, meaning air flow is dependent upon thermal buoyancy, movement of the vehicle itself, and the wind speed. To control the internal trailer environment, the National Pork Board’s Transport Quality Assurance (TQA) program recommends that pigs (>27 °C) and bedding (>15 °C) are sprinkled to facilitate evaporative cooling with the intention of reducing heat stress [6]. However, these recommendations are based on experiential information rather than scientific data [6]. Therefore, the objectives of these experiments were to compare the effects of sprinkling methods inside trailers on (1) market weight pig measures at the time of unloading and (2) transport losses at the plant.

## 2. Experimental Section

### 2.1. General Procedures for Both Experiments

The protocol for these experiments was approved by the Iowa State University Institutional Animal Care and Use Committee and data was collected during 3 weeks in July 2012.

#### 2.1.1. Animals, Farms, and Handling

The company’s loading crew sorted and moved market weight barrows and gilts from their home pen to the entrance of the loading ramp. The trucker moved the pigs up the loading ramp and onto the trailer. Both the loading crew and the trucker used a combination of sort boards, rattle paddles, and electric prods during loading (the number of times these devices were used was not recorded for these experiments). 

Following TQA recommendations, any pig that became hot or stressed during loading would not have been loaded onto the truck. However, no pigs in these experiments met that criteria. The pigs were transported from commercial finishing facilities to a commercial processing plant, all located in Iowa. Transport occurred throughout the day and night. The trucker unloaded the pigs and plant personnel moved the pigs from the loading dock to the rest pens. During unloading, plant personnel and the trucker used paddles, rattles, and boards (the number of times these devices were used was not recorded for these experiments). 

#### 2.1.2. Treatments and Experimental Design

Treatment one (control) was defined as not sprinkling pigs or bedding on the trailer. Treatment two (pigs only) was defined as pigs being sprinkled after loading was completed for 6 to 8 min. Treatment three (bedding only) was defined as bedding being sprinkled 4 to 6 min before the start of loading. Treatment four (pigs and bedding) was defined as both pigs and bedding being sprinkled for 6 to 8 min. Due to concerns about pig well-being, the control treatment was not applied when the temperature was ≥26.7 °C. Therefore, two data sets will be presented: WARM (temperature <26.7 °C; 4 treatments) and HOT (≥26.7 °C; 3 treatments) All treatments were applied by the researchers and were randomly assigned to trailers. 

#### 2.1.3. Transport Trailers and Density

All pigs were transported on aluminum drop deck (pot belly) trailers 17 m in length with diamond plate flooring. These were owned and operated by drivers employed by trucking companies contracted through the plant. 

All compartments in the trailer were stocked according to the industry’s current standard operating procedure of 0.41 m^2^/pig or ~171 pigs/load [6]. The plant provided data on the number of pigs/trailer and the average weight of pigs on a trailer. For these experiments a density value was calucated and added to the statistical model because previous work has found density is an important variable in affecting animal based measures and transport losses [7,8,9].

Density = [(average pig weight per trailer) × (pigs per trailer)] / (m^2^ floor space in trailer)

#### 2.1.4. Temperature Humidity Index

Ambient relative humidity and air temperature were measured at an airport 16.9 km from the plant. The airport data logger (1088 Hygrothermometer, Technical Service Laboratory, Fort Walton Beach, FL, USA) collected temperature and dew point. Relative humidity was then calculated from dew point and temperature measurements. The airport data logger was accurate for ambient temperature and for dew point ± 0.003 °C. Ambient temperature (T) and relative humidity (RH) were used to calculate a temperature humidity index (THI) using the following equation [10]:

THI = T − {[0.55 − (0.0055 × RH_decimal_)](T − 14.5)}

This equation was found to fit the model of pig transport by Fitzgerald and others [7]. Additionally, other evidence suggests that it is important to consider both temperature and humidity when determining heat stress in pigs [11,12,13]. 

### 2.2. Experiment 1: Effects of Sprinkling Inside Trailers Transporting Market Weight Pigs During WARM and HOT Weather on Pig Measures and Bedding Moisture at Unloading

A total of 51 loads were used in WARM- and 86 loads were used in HOT weather. In WARM weather, the treatments were control (n = 24), pigs only (n = 13), bedding only (n = 7), and pigs and bedding (n = 7). In HOT weather, the treatments were pigs only (n = 41), bedding only (n = 18), and pigs and bedding (n = 27). 

#### 2.2.1. Pig Measures

Pig measures were collected on a random sample of pigs at unloading using live observation. A random sample of pigs was defined as ignoring the first ~10 pigs at the beginning of unloading, counting 50 pigs (group A), ignoring a further ~10 pigs, and counting another 50 pigs (group B). This provided 100 pigs/load. For groups A and B, the following pig measures were tallied: vocalizations, slips and falls, and stress signs. Vocalizations were defined as an extended sound of high amplitude and frequency produced with an open mouth [2]. Slips were defined as a knee or hock touching the ground; falls were defined as a pig’s body touching the ground [14]. Slips and falls were tallied as a single measure. Stress signs were defined as open mouth breathing, muscle tremors, and red-blotchy skin [15]. Surface temperature was measured laterally near the midline on 5 randomly selected pigs from groups A and B (total of 10 pigs/load). Surface temperature was measured with a dual laser infrared thermometer (model 42570, Extech Instruments, Nashua, NH, USA) which was accurate to ±1 °C. 

#### 2.2.2. Bedding Moisture

A total of 8 fresh bedding samples and 140 used bedding samples were collected from trailers. Fresh samples (0 loads) were defined as bedding that had not been previously used for transporting pigs. A fresh bedding sample was ~45 g. After each trailer had unloaded at the plant, a used bedding sample, defined as bedding which had transported ≥ 1 trailer loads of pigs was collected. Half of the used bedding was collected from the bottom trailer deck and the remainder was collected from the top deck. Each used bedding sample was ~410 g. Bedding samples were stored in a cardboard box at room temperature (~21 °C) on the floor for no longer than 1 wk after trial completion. 

Bedding moisture was determined following standard operating procedure for drying samples. A tin measuring 7.6 cm wide by 2.2 cm deep (model A90, Wilkinson Industries Inc., Fort Calhoun, NE, USA) was weighed. Each bedding sample was kneaded by hand inside the closed storage bag for ~30 s. Two, 3 to 6 g subsamples (subsample A and B) were removed from the bag using a spoon. Subsample A was placed in one tin and subsample B was placed in a second. The bedding subsample in its respective tin was weighed (accurate ± 0.03 mg; model AT261 DeltaRange, Metler-Toledo GmBh Laboratory and Weighing Technologies, Greifensee, Switzerland) to determine wet weight. Bedding subsamples were dried for ~20 to 24 h at 100 °C in a convection oven (model DKN810, Yamato Scientific America Inc., Santa Clara, CA, USA). After drying, subsamples were re-weighed; this was defined as the dry weight. Moisture percent for each subsample was calculated using the following equation [16]:

Moisture percent = [(dry weight) / (wet weight)] × 100

A standard deviation of moisture percentage between subsample A and subsample B and an average of the moisture percent of subsample A and subsample B were calculated. Between subsample A and subsample B, the coefficient of variation (CV) was calculated using the following equation:

CV = (Standard deviation / average) × 100

If the CV ≥ 10 the sample was re-subsampled and dried a second time (n = 14). If the sample was still found to be too variable on the second drying, that samples were removed from the data set (n = 0). The data from bedding moisture will be presented descriptively separated by the number of loads on the bedding, ranging from 0 to ≥ 4 loads. 

#### 2.2.3. Transport Events

Researchers recorded the time that loading started and ended, the time the trailer left the farm, and the time that unloading started and ended. Processing plant records provided the arrival time of the trailer at the plant. Loading was defined as the time interval from the first pig’s front foot stepped onto the trailer until the last pig’s hind foot stepped onto the trailer. Wait time at the farm was defined as the time from when the last pig’s hind foot stepped onto the trailer until the trailer left the farm. Transport was defined as the time interval from when trailer left the farm until the trailer arrived at the plant. Wait time at the plant was defined as the time interval from when the trailer arrived at the plant until the first pig’s front foot stepped off the truck. Unloading was defined as the time interval from when the first pig’s front foot stepped off the truck until the last pig’s hind foot stepped off the truck. Total transit time was defined as the time from when the first pig’s front foot stepped onto the trailer (start of loading) until the last pig’s hind foot stepped off the trailer (end of unloading). 

#### 2.2.4. Bedding Level

The number of 0.2 m^3^ (22.7 kg) bags of wood shaving bedding/trailer were recorded. Because trailers rely on passive ventilation, in the winter bedding is believed to insulate pigs from extreme cold. In the summer, less bedding is included as a means of providing traction and absorbing waste. 

### 2.3. Experiment 2: Effects of Sprinkling Inside Trailers on Market Weight Pig Transport Losses During WARM and HOT Weather

A total of 82 loads were used in WARM- and 54 loads were used HOT weather to determine if sprinkling effected transport losses. In WARM weather, the treatments were control (n = 48), pigs only (n = 11), bedding only (n = 15), and bedding and pigs (n = 8). In HOT weather, the treatments were pigs only (n = 31), bedding only (n = 9), and bedding and pigs (n = 14). 

#### Transport Losses at the Plant

Plant employees identified non-ambulatory (sum of fatigued and injured) [2] and dead (sum of euthanized- and dead on arrival), Total transport losses were defined as the sum of non-ambulatory and dead.

### 2.4. Statistical Analysis

For both experiments, data were evaluated for missing and erroneous values by using the filter feature in Excel (Microsoft Office 2010, Microsoft Redmond, WA, USA). The remaining analyses were completed using SAS software (SAS V 9.2 Institute Inc., Cary, NC, USA). Using the means and sort procedures data was checked for erroneous and potential outlier data points. Data that was identified as a potential outlier was checked against the original data. If correct it was simply highlighted in the excel data, if incorrect that value was substituted per the original data. Because all four treatments were only present when the temperature was <26.7 °C SAS was used to create two data sets from the single excel file data was originally entered (WARM and HOT data sets). *p* < 0.05 was considered significant for both experiments. *p* ≤ 0.10 was considered tending for both experiments. For both experiments, each variable collected was evaluated on whether it should be present in the model. Variables that might have affected the pig measures were attempted in the model. Those variables that were found to be significant or were indicated by previous research to cause variation in pig transport were retained for the final model.

#### 2.4.1. Experiment 1: Effects of Sprinkling Inside Trailers Transporting Market Weight Pigs During WARM and HOT Weather on Pig Measures and Bedding Moisture at Unloading

Because researchers sometimes counted more, or less than 50 pigs/group, data for vocalizations, slips and falls, and stress signs were analyzed as a percent of the pigs counted:

Percent pig measure = [(number of times a measure was counted) / (number pigs counted in that group)] × 100

Furthermore, the SAS program (SAS 9.3, SAS Institute Inc., Cary, North Carolina) was used to create a new variable from the percent of vocalizations, slips and falls, and stress signs from group A and group B of 50 (e.g., [percent stress signs group A + percent stress signs group B]/2). Surface temperature was analyzed as an average of the 10 pigs measured per load. 

Data were analyzed using a mixed model (PROC MIXED, SAS 9.3 SAS, Institute Inc., Cary, NC, USA) where the response variables, surface temperature, vocalizations, slips and falls, and stress signs, were analyzed using sprinkling treatment and bedding level as fixed effects, THI at unloading and density as linear covariates, and farm, trucking company, and researcher at the plant as random effects.

#### 2.4.2. Experiment 2: Effects of Sprinkling Inside Trailers on Market Weight Pig Transport Losses During WARM and HOT Weather

Analysis of non-ambulatory-, dead-, and total transport losses per trailer was performed using a generalized linear mixed model (GLIMMIX procedure, SAS 9.3 Cary, NC, USA). The data approximated a Poisson distribution and was log transformed by the GLIMMIX procedure (SAS 9.3 Cary, NC, USA) prior to statistical analysis. The model used sprinkling treatment as a fixed effect, THI at loading and density as linear covariates, and farm and trucking company as random effects. The ILINK option (SAS 9.3 Cary, NC, USA) was used to back-transform least squares means into their original unit of measure for ease of interpretation.

## 3. Results and Discussion

### 3.1. Experiment 1: Effects of Sprinkling Inside Trailers Transporting Market Weight Pigs During WARM and HOT Weather on Pig Measures and Bedding Moisture at Unloading

#### 3.1.1. Sprinkling

In WARM weather, sprinkling treatment had no observed effect on surface temperature, vocalizations, or slips and falls (*p* ≥ 0.18). However, stress signs were 2% greater for the bedding and pig treatment than control (*p* = 0.03; Table 1). In HOT weather, sprinkling method had no effect on surface temperature, vocalizations, slips and falls, or stress signs (*p* ≥ 0.19; Table 1).

**Table 1:** Experiment 1. Effects of sprinkling ^1^ on pig measures ^2^ in market weight pigs in WARM ^3^ and HOT ^4^ weather.

	Sprinkling treatment					
WARM weather, measure	Control	Pigs only	Bedding only	Bedding and pigs	*p*-value	R^2^
	n = 24	n = 13	n = 7	n = 7		
Surface temperature, ° C	32.2 ± 0.5	32.7 ± 0.4	33.1 ± 0.6	32.3 ± 0.6	0.18	0.41
Vocalizations, % of pigs counted	2.4 ± 1.8	2.6 ± 1.8	2.7 ± 1.9	3.4 ± 1.9	0.65	0.04
Slips and falls, % of pigs counted	0.7 ± 0.2	0.5 ± 0.2	0.5 ± 0.3	0.2 ± 0.3	0.61	0.10
Stress signs, % of pigs counted	0.6 ± 0.4 ^a^	0.5 ± 0.4 ^a,b^	1.5 ± 0.6 ^a,b^	2.6 ± 0.6 ^b^	0.03	0.03
**HOT weather**	**n = 0**	**n = 41**	**n = 18**	**n = 27**		
Surface temperature, ° C	.	35.3 ± 0.3	34.8 ± 0.3	34.9 ± 0.3	0.19	0.37
Vocalizations, % of pigs counted	.	1.7 ± 1.2	1.7 ± 1.2	2.0 ± 1.2	0.63	0.01
Slips and falls, % of pigs counted	.	0.6 ± 0.4	0.9 ± 0.4	0.6 ± 0.4	0.51	0.05
Stress signs, % of pigs counted	.	7.2 ± 1.4	6.0 ± 1.6	5.7 ± 1.4	0.35	0.31

^1^ Sprinkling methods, applied by researchers were: bedding only (bedding already being damp or being watered down by the researcher for 4–6 min before the start of loading), pigs only (pigs being watered after loading completed for 6–8 min when the bedding on the trailer was dry before loading started), pigs and bedding (both pigs and bedding being watered). ^2^ Pig measures were: surface temperature (measured laterally near the midline with a dual laser infrared thermometer on 10 pigs/load), vocalizations (an extended sound of high amplitude and frequency produced with an open mouth [2], slips (a knee or hock touching the ground) and falls (a pig’s body touching the ground [14]) and stress signs (open mouth breathing, muscle tremors, and red-blotchy skin [15]). ^3^ Warm weather was defined as the temperature < 26.7 °C; based on 51 loads. ^4^ Hot weather was defined as the temperature ≥ 26.7 °C; based on 86 loads. ^a,b,c^ Values within the same row without common superscripts differed (*p* ≤ 0.05).

Pig vocalizations are a non-invasive measure and which may indicate the distress [17,18,19]. Kiley [17] has described 13 different types of pig vocalizations being expressed at different ages and within a variety of situations, for example social-greeting or non-social-startle. Furthermore, studies have found that squeal type vocalizations are associated with unpleasant situations [17,18,20]. Although vocalizations were not different in the context of this study between bedding levels, they may still be a useful non-invasive measure when assessing how pigs are coping with the transport process. 

**Figure 1:**
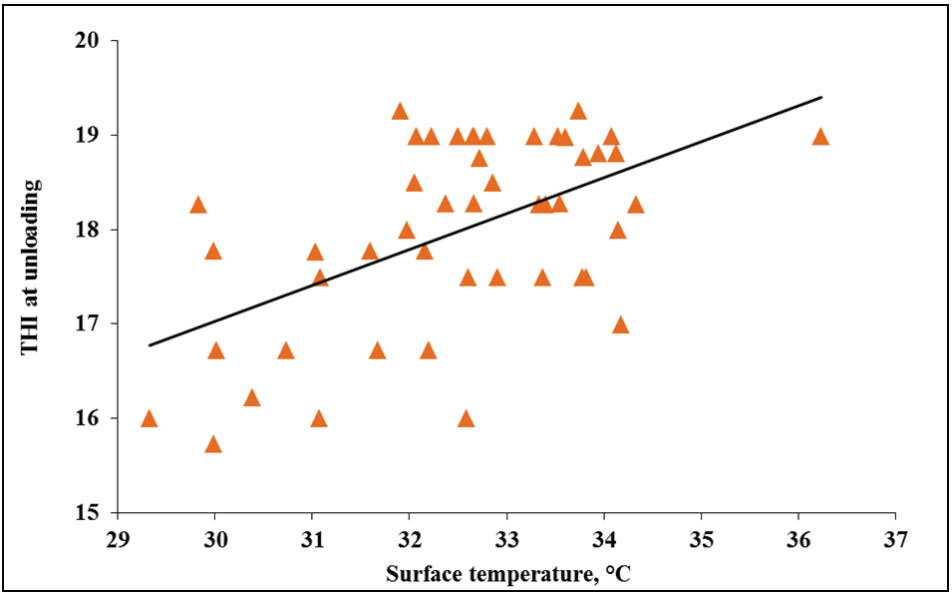
Experiment 1. Effects of temperature humidity index (THI) at unloading on surface temperature of market weight pigs at unloading in WARM weather (<26.7 °C; *p* < 0.01, R^2^ = 0.41).

**Figure 2:**
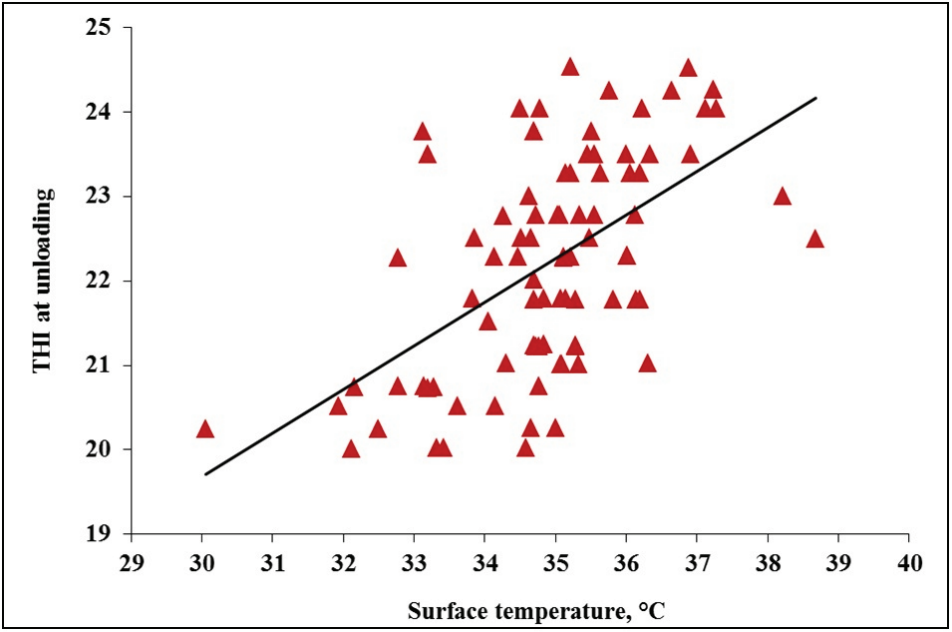
Experiment 1. Effects of THI at unloading on surface temperature of market weight pigs at unloading in HOT weather (≥26.7 °C; *p* < 0.01, R^2^ = 0.35).

The current study observed surface temperature ranging 29.3 °C to 36.2 °C in WARM weather and 30.1 °C to 38.7 °C in HOT weather (Figure 1 and Figure 2). Although past research reported surface temperature for market weight pigs ranging between 38.6 °C to 39.5 °C [21,22], a review by Fox [23] in Canada reported that sprinkled pigs had 10% lower surface temperature than those pigs which were not sprinkled. Based on the surface temperatures seen in this study, it seems pigs in this study were not heat stressed and in turn this may indicate that the pigs’ physiological responses combined with the sprinkling treatments could be effective at mitigating heat stress.

#### 3.1.2. Bedding Moisture

Fresh bedding averaged ~5% moisture before any sprinkling treatments were applied. After sprinkling treatments had been applied and one load of pigs had been transported to the plant, bedding only and bedding and pigs sprinkling treatments had ~5% more moisture than pigs only or control treatments. However, when ≥2 loads had been transported, bedding moisture held constant at ~65% regardless of sprinkling treatment (Table 2). 

**Table 2:** Experiment 1. Bedding moisture ^1^ by sprinkling method ^2^ combined WARM ^3^ and HOT ^4^ weather.

	Sprinkling treatment							
	Control		Bedding only		Pigs only		Bedding and pigs	
	Bedding moisture (%)							
Loads	Mean	SD	Mean	SD	Mean	SD	Mean	SD
0	5.5	1.5	3.7	.	7.6	5.3	4.5	.
1	63.3	6.2	68.8	8.2	62.5	15.2	70.6	8.7
2	63.2	5.4	65.4	5.2	63.6	11	73.1	9.4
3	62.5	4.2	59.4	6.4	70.1	9.4	62.8	8.3
≥4	60.6	2	61.7	7.3	70.7	5	68.4	5.3

^1^ There were 27 samples collected from trailers given the control treatment: 0 loads, n = 2; 1 load, n = 14; 2 loads, n = 6; 3 loads, n = 2; and ≥4 loads, n = 3. There were 58 samples taken from trailers given the pigs only treatment: 0 loads, n = 1; 1 load, n = 26; 2 loads, n = 11; 3 loads, n = 11; and ≥4 loads, n = 6. There were 25 samples collected from trailers given the bedding only treatment: 0 loads, n = 1; 1 load, n = 6; 2 loads, n = 9; 3 loads, n = 5; and ≥4 loads, n = 4. There were 38 samples taken from trailers given the pigs and bedding treatment: 0 loads, n = 1; 1 load, n = 12; 2 loads, n = 10; 3 loads, n = 6; and ≥4 loads, n = 9. Bedding moisture was calculated by: [(dry bedding weight)/(wet bedding weight)] × 100. ^2^ Sprinkling treatments were defined as: control (no water on pigs, bedding dry), pigs only (bedding dry, pigs watered for 6–8 min), bedding only (bedding already wet or bedding watered for 4–6 min), and bedding and pigs (both pigs and bedding wetted as previously described). ^3^ WARM weather was defined as <26.7 °C; based on 50 loads. ^4^ HOT weather was defined as ≥26.7 °C; based on 92 loads.

Lack of increasing moisture with subsequent loads suggests that only fresh bedding is effective at moisture absorbance. This study only observed trailers with wood shavings. Wood shavings are less absorbent than straw or corn stover (1.15 *vs.* 1.97 *vs.* 2.70 mean absorbency factor, respectively; [24,25]. Data from this study supports the TQA guidelines, suggesting trailers should be washed out and fresh bedding applied after every load [6], which may also help with better traction for the pigs [26], improve biosecurity [27] and may decrease stress [28,29,30]. However, individual company protocols vary on washout frequency and application of fresh bedding. Trailer wash out can range from $15 to $190 [31]. Annually, washing out trailers between each load and re-bedding the trailer has been estimated to cost ~$8 to $108 million annually [32]. However, this estimate does not include potential lost income to the driver while washing the trailer or environmental implications for water usage and bedding disposal. In addition, using three instead of six bags in warm weather (defined as temperature ranging 16.1 °C to 43.4 °C) has been estimated to save $13 million [32]. Hence, adding the cost of washout, Kephart and others [32] found that using three bags/trailer and washing out after every load would cost between ~$22 and $121 million annually. A cost benefit analysis for using fresh bedding after every load, in relation to overall swine well-being improvements is suggested. 

#### 3.1.3. Transport Events

The mean loading times in this study (Table 3) were similar to previous studies by Gesing and others [15] and Brown and colleagues [33] at 38 min and 45 min respectively. Mean unloading time in the current study was also similar to Gesing and others [15] at 18 min. Transport time was about double for that reported by Gesing and others (59 min [15]), but comparable to Pilcher and others** (107 min [34]). A possible explanation for this increased transport time in the current study was the distance between the farms and the plant (ranging from 74 to 296 km). The plant used by Gesing and others [15] was 85 km from the farm. The wait time at the plant observed in this study was longer than that reported by Gesing and others (9 min [15]). However, Pilcher and colleagues [34] and Gesing and colleagues [35] reported a mean wait time of ~21 min. Wait time can be affected by a variety of factors such as time of arrival and labor availability at the plant. A wait of ~20 min is very acceptable for the U.S. swine industry and within the context of this study was not detrimental to pig well-being. Transport event times will need to be carefully reviewed by trucking companies, processing facilities, and the truckers themselves due to changes made by the U.S. Department of Transportation (DOT). As of July 1st, 2013, the DOT hours-of-service safety regulation states that after 8 h of driving the trucker must take a 30 min break away from the truck [36]. For transportation of non-animate goods this will likely not be a challenge. However, if live animals are being transported, increased time when the trailer is stationary can result in an increased in both temperature and relative humidity [37].

**Table 3:** Experiment 1. Descriptive statistics for transport events ^1^ for sprinkling method in market weight pigs for both WARM ^2^ and HOT ^3^ weather.

WARM weather; Event, min	Mean	SD ^4^	Min ^5^	Max ^6^
Loading	32	12	14	65
Wait time at farm	7	6	2	42
Transport	156	43	63	280
Wait time at plant	15	13	1	50
Unloading	15	6	5	36
Total time	230	52	126	390
**HOT weather**				
Loading	28	11	13	65
Wait time at farm	9	4	1	19
Transport	168	41	48	255
Wait time at plant	24	15	5	65
Unloading	24	15	5	65
Total time	238	62	56	369

^1 ^Transport events were loading (the time from when the first pig stepped on to the trailer until the last pig stepped onto the trailer), wait time at the farm (the time from when the last pig stepped onto the trailer until the trailer left the farm), transport (the time from when the trailer left the farm was closed until the truck arrived at the plant), wait time at the plant was defined as the time from when the truck arrived at the plant until the first pig stepped off), unloading (the time from the first pig stepped off the trailer until the last pig stepped off the trailer the trailer). ^2^ WARM weather was defined as <26.7 °C; based on 50 loads. ^3^ HOT weather was defined as ≥26.7 °C; based on 92 loads. ^4^ SD abbreviation for standard deviation. ^5^ Min abbreviation for minimum. ^6^ Max abbreviation for maximum.

#### 3.1.4. Temperature Humidity Index

In WARM and HOT weather, with increasing THI at unloading pig surface temperature increased (*p* < 0.01; Figure 1 and Figure 2). In WARM weather, as THI increased from ~17 to 19, surface temperature increased ~7 °C. In HOT weather, as THI increased from ~20 to 24, surface temperature increased ~9 °C.

In WARM weather, no THI effects were observed at unloading on vocalizations, slips and falls, or stress signs (*p* = 0.19, R^2^ = 0.04; *p* = 0.15, R^2^ = 0.10; and *p* = 0.44, R^2^ = 0.03, respectively; data not presented). In HOT weather, there were no THI observed effects on vocalizations or slips and falls (*p* = 0.96, R^2^ = 0.01; *p* = 0.40, R^2^ = 0.05; data not presented). 

**Figure 3:**
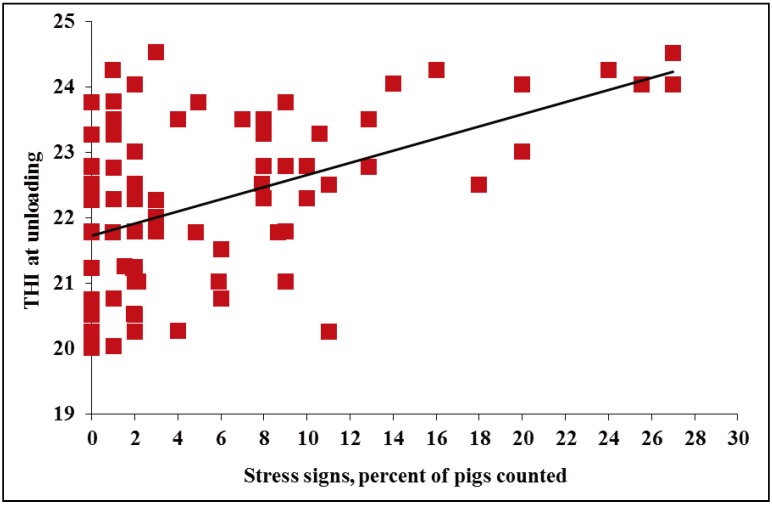
Experiment 1. Effects of THI at unloading on stress signs of market weight pigs at unloading in HOT weather (≥26.7 °C; *p* < 0.01, R^2^ = 0.31).

However, it was observed in HOT weather that as THI increased from ~20 to 24, stress signs at unloading increased ~27% (*p* < 0.01, Figure 3). Increased stress signs, such as open mouth breathing and red blotchy skin, could be explained by the pig’s natural heat coping mechanisms. Although surface temperature ranges seen in this study are not reflective of severely heat stressed pigs this may simply mean that their physiological mechanisms were acting effectively. It is difficult to speculate as to why an increase in THI would increase slips and falls. It may be that pigs are motivated to exit the trailer quicker and hence lose their footing more because of the heat in the trailer. However, this theory would need to be further evaluated in controlled heat and behavioral studies.

#### 3.1.5. Density

In WARM weather, no density effects were observed on surface tempearture, vocalizations, slips and falls, or stress signs (*p* = 0.45, R^2^ = 0.41 and *p* = 0.39, R^2^ = 0.04; *p* = 0.15, R^2^ = 0.10; and *p* = 0.98, R^2^ = 0.03, respectively; data not presented). In HOT weather, no effects of density were observed on surface temperature or vocalizations (*p* = 0.74, R^2^ = 0.37; and *p* = 0.36, R^2^ = 0.01, respectively; data not presented). 

In HOT weather as density increased from ~275 to 300 kg/m^2^, slips and falls tended to decrease ~5% (*p* = 0.07, R^2^ = 0.10; data no presented) and stress signs tended to also decrease ~27% (*p* = 0.07, R^2^ = 0.03; data no presented). Pigs in the current study were transported at an average density of 297 and 294 m^2^/pig in WARM and HOT weather respectively, but the density equation used factored in weight and number of pigs on the trailer and was presented as a continous variable. This may be why the stress results in the current work disagree with Ritter and others [9] who reported that pigs transported at 0.52 m^2^/pig (~252 kg/m^2^) had a higher incidence of skin discoloration than pigs transported at 0.39, 0.42, or 0.46 m^2^/pig (~336, 312, and 285 kg/m^2^ respectively). Direct comparisons for changes in pig surface temperature based on density have not been published. Ritter and others [38] reported that density on the trailer had no effect on rectal temperature between 265 kg/m^2^ (0.39 m^2^/pig) and 333 kg/m^2^ (0.49 m^2^/pig) kg/m^2^ respectively. Chung and others noted as rectal temperature increased surface temperature increased in a linear manner [21]. This raises an interesting statistical discussion in regards to fixed effects and covariates, the use of both density and THI equations and in turn results, making comparison of these data sets challenging. 

#### 3.1.6. Bedding Level

In WARM weather, no effects of bedding were observed on surface temperature, vocalizations, slips and falls, or stress signs (*p* ≥ 0.12; Table 4). In HOT weather, no effects of bedding were observed on vocalizations or slips and falls (*p* ≥ 0.28; Table 4). However, in HOT weather, increasing bedding from two to three bags/trailer increased surface temperatures 0.6 °C and stress signs 2.5 % (*p* ≤ 0.05; Table 4). This may indicate that in hot weather extra bedding may exacerbate heat stressed experienced by pigs on the trailer.

**Table 4:** Experiment 1. Effects of bedding level ^1^ on pig measures ^2^ in market weight pigs in WARM ^3^ and HOT ^4^ weather.

WARM weather; measures	Bedding level		*p*-value	R^2^
	3	4		
	n = 41	n = 10		
Surface temperature, °C	32.3 ± 0.4	32.9 ± 0.6	0.12	0.41
Vocalizations, % of pigs counted	3.0 ± 1.7	2.6 ± 1.8	0.59	0.04
Slips and falls, % of pigs counted	0.5 ± 0.2	0.4 ± 0.3	0.72	0.10
Stress signs, % of pigs counted	1.1 ± 0.3	1.5 ± 0.5	0.42	0.03
**HOT weather**	**n = 67**	**n = 19**		
Surface temperature, °C	34.7 ± 0.2	35.3 ± 0.3	0.05	0.37
Vocalizations, % of pigs counted	1.9 ± 1.2	1.7 ± 1.2	0.56	0.01
Slips and falls, % of pigs counted	0.7 ± 0.4	0.6 ± 0.4	0.77	0.05
Stress signs, % of pigs counted	5.1 ± 1.3	7.6 ± 1.6	0.03	0.31

^1^ Bedding level is the number of ~0.2 m^3^ (22.7 kg) bags of wood shavings/trailer. ^2^ Pig measures were: surface temperature (measured laterally near the midline with a dual laser infrared thermometer on 10 pigs/load), vocalizations (extended sounds of high amplitude and frequency produced with an open mouth [2]), slips and falls (a knee, hock, or body touching the ground [14]), and stress signs (open mouth breathing, muscle tremors, and red-blotchy skin [15]). ^3^ WARM weather was defined as the temperature <26.7 °C; based on 48 loads. ^4^ HOT weather was defined as the temperature ≥26.7 °C; based on 88 loads.

### 3.2. Experiment 2: Effects of Sprinkling Inside Trailers on Market Weight Pig Transport Losses During WARM and HOT Weather

#### 3.2.1. Sprinkling

In WARM weather, the one non-ambulatory pig was from a trailer allocated to the pigs and bedding sprinkling treatment. In WARM and HOT weather, no effect of sprinkling treatment was observed for non-ambulatory, dead, or total transport losses (*p* ≥ 0.18; Table 5). It is important to note that total transport losses in the present study were ~0.17 pigs/trailer or ~0.10%. Additionally, when comparing the total transport loses percentages from the present study with losses on a national level, the estimated national average for total losses was 0.69% [38]. If a higher rate of losses were seen in this study there may have been bigger differences between the treatments allowing for significance. 

**Table 5:** Experiment 2. Effects of sprinkling ^1^ on transport losses ^2^ in market weight pigs in WARM ^3^ and HOT ^4^ weather.

WARM weather; transport losses, pigs/trailer	Sprinkling Treatment				*p*-value	R^2^
	Control	Pigs only	Bedding only	Pigs and bedding		
	n = 48	n = 11	n = 15	n = 8		
Non-ambulatory	.	.	.	.	.	.
Dead	0.06 ± 0.04	0.13 ± 0.11	0.11 ± 0.09	0.00 ± 0.00	0.76	0.01
Total transport losses	0.06 ± 0.04	0.24 ± 0.15	0.13 ± 0.10	0.00 ± 0.01	0.33	0.03
**HOT weather**	**n = 0**	**n = 31**	**n = 9**	**n = 14**		
Non-ambulatory	.	0.07 ± 0.04	0.00 ± 0.00	0.01 ± 0.02	0.28	0.32
Dead	.	0.37 ± 0.10	0.21 ± 0.14	0.16 ± 0.09	0.31	0.27
Total transport losses	.	0.45 ± 0.11	0.18 ± 0.12	0.19 ± 0.10	0.18	0.35

^1^ Sprinkling methods, applied by researchers were: Control (no water sprinkled and bedding dry; not applied in HOT weather), bedding only (bedding already being damp or wetted for 4–6 min before the start of loading), pigs only (pigs being wetted after loading completed for 6–8 min when the bedding was dry), pigs and bedding (both pigs and bedding being watered). ^2^ Transport losses were non-ambulatory (sum of fatigued and injured pigs), dead (sum of euthanized- and dead on arrival), and total transport losses (sum of non-ambulatory and dead). ^3^ WARM weather was defined as the temperature <26.7 °C; based on 79 loads. ^4^ HOT weather was defined as the temperature ≥26.7 °C; based on 49 loads.

#### 3.2.2. Temperature Humidity Index

In WARM weather, the one non-ambulatory pig occurred when THI was 18. In WARM weather, no THI at loading effects were observed were observed on dead or total transport losses (*p* = 0.94, R^2^ = 0.01; *p* = 0.90, R^2^ = 0.03; data not presented). In HOT weather, no THI effects were observed at loading on non-ambulatory, dead, or total transport losses (*p* = 0.66, R^2^ = 0.32; *p* = 0.12, R^2^ = 0.27; *p* = 0.19, R^2^ = 0.35; data not presented). Pigs in the current study were only transported in the summer months. This may explain why the results differ from Fitzgerald and colleagues [7] who observed more pigs than the current study in all seasons. Fitzgerald and colleagues reported [7] increased total transport losses as both THI and density increased. Additionally, they observed higher numbers of transport losses (1.41 total transport losses pigs/trailer) than were observed in the current study (in WARM 0.14 total transport losses pigs/load; in HOT weather 0.25 total transport losses pigs/load). It is difficult to compare the results found in the current study with other studies because other studies use temperature and relative humidity separately rather than in as an index [8,34,38,39].

#### 3.2.3. Density

**Figure 4:**
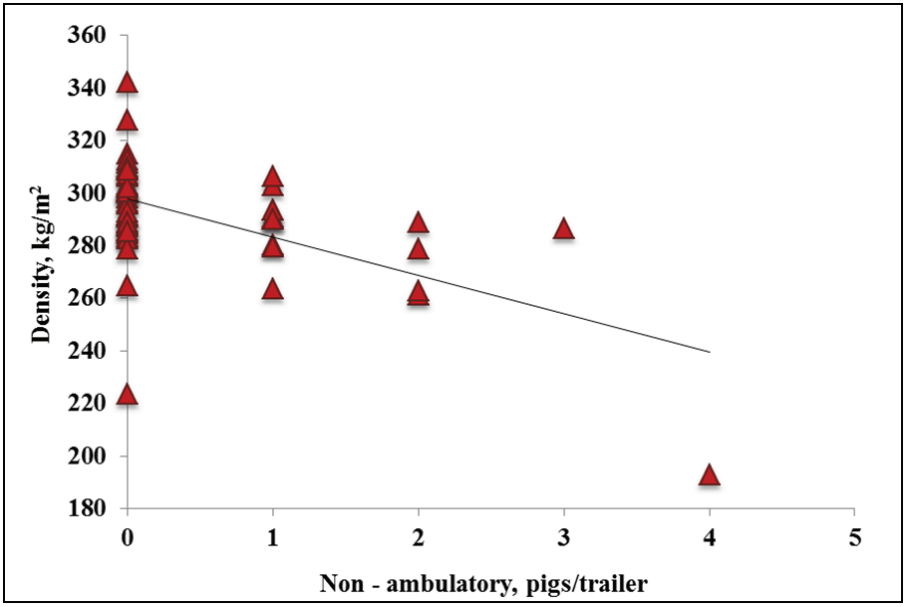
Experiment 2. Effects of density on trailers on non-ambulatory pigs per trailer in market weight pigs at unloading in HOT weather (≥26.7 °C; *p* = 0.01, R^2^ = 0.32).

**Figure 5:**
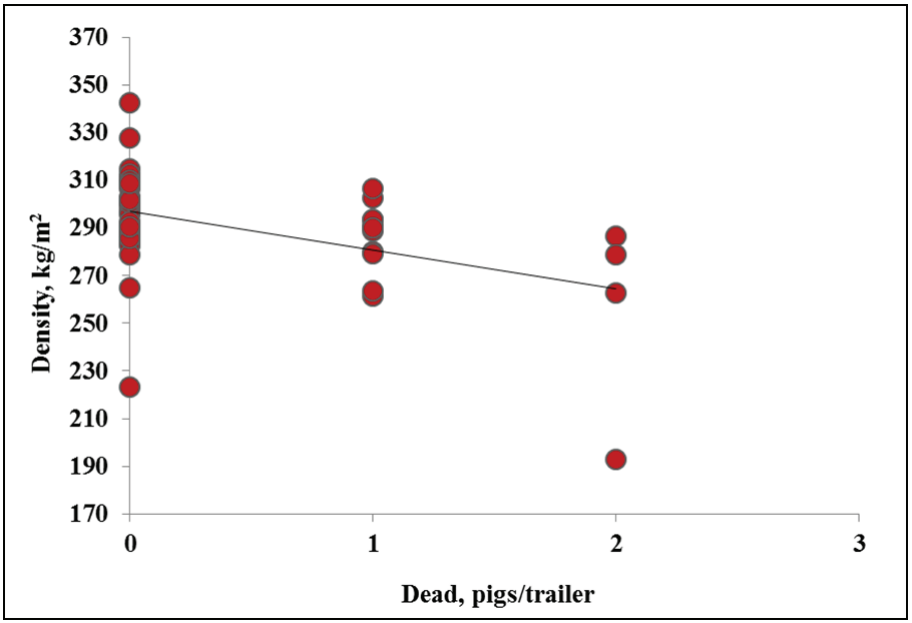
Experiment 2. Effects of density on trailers on dead pigs per trailer in market weight pigs at unloading in HOT weather (≥26.7 °C; *p* = 0.01, R^2^ = 0.27).

**Figure 6:**
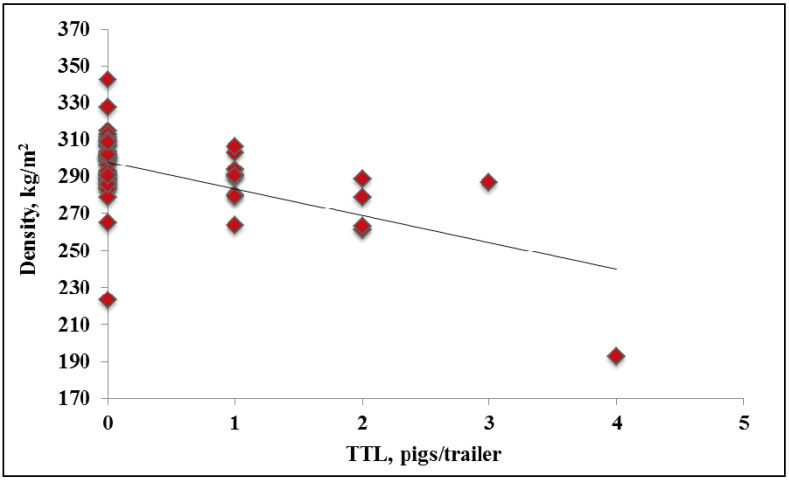
Experiment 2. Effects of density on trailers on total transport losses pigs per trailer in market weight pigs at unloading in HOT weather (≥26.7 °C; *p* < 0.01, R^2^ = 0.36).

In WARM weather, the one non-ambulatory occurred on a trailer with a density of 291 kg/m^2^. In WARM weather, no effects of density were observed on dead or total transport losses (*p* = 0.86, R^2^ = 0.01; *p* = 0.81, R^2^ = 0.03; data not presented). In HOT weather, no effects of density were observed on non-ambulatory pigs (*p* = 0.01, R^2^ = 0.32; Figure 4). In HOT weather, decreasing density from ~300 to 265 kg/m^2^ increased dead pigs/trailer by 2 (*p* = 0.01; Figure 5). In HOT weather, decreasing density from ~300 to 240 kg/m^2^ increased total transport losses by four pigs/trailer (*p* < 0.01; Figure 6). However the relationship between both dead and total transport losses and density was weak (R^2^ = 0.27 and R^2^ = 0.35, respectively). It is difficult to explain why dead and total transport losses increased with decreasing density, but pigs in this study were transported at similar density ranges compared to previous studies [7,8,9]. 

The current study reviewed a relatively small number of loads compared to past studies [7,8] and also observed a lower rate of transport losses [7,8,9] than previous work. This may explain why the results in the current study disagree with past studies. Pilcher and colleagues [34] found no effects of density on dead or total losses in pigs transported in November, December, May, and June. However, Pilcher and colleagues used density as a treatment and it was fixed rather than continous. Fitzgerald and colleagues [7] predicted that total transport losses would increase constantly as density increased. Ritter and colleagues [8] found that increasing density increased non-ambulatory and total transport losses. Ritter and others [9] reported increasing density increased non-ambulatory and total transport losses.

## 4. Conclusions

Stressors during transportation have been shown to be additive [7,40]. Therefore, reducing or preventing stressors may improve pig well-being. A variety of factors may influence animal based measures indicative of well-being and transport losses in the market weight pigs. Although the current study did not find any observed sprinkling effects for pig measures or transport losses it is extremely important to note that the inference space of this study is relatively small (July in Iowa), so further studies should be conducted to see if these results are applicable to other geographical regions and seasons. 
